# PPARγ as an E3 Ubiquitin-Ligase Impedes Phosphate-Stat6 Stability and Promotes Prostaglandins E_2_-Mediated Inhibition of IgE Production in Asthma

**DOI:** 10.3389/fimmu.2020.01224

**Published:** 2020-06-19

**Authors:** Jia Wu, Yan Wang, Yu Zhou, Yuqing Wang, Xiaowan Sun, Ye Zhao, Youfei Guan, Yu Zhang, Wei Wang

**Affiliations:** ^1^Department of Immunology, School of Basic Medical Sciences, Peking University, NHC Key Laboratory of Medical Immunology (Peking University), Beijing, China; ^2^State Key Laboratory of Bioactive Substance and Function of Natural Medicines, Institute of Materia Medica, Chinese Academy of Medical Sciences and Peking Union Medical College, Beijing, China; ^3^Center of Basic Medical Research, Institute of Medical Innovation and Research, Peking University Third Hospital, Beijing, China; ^4^Advanced Institute for Medical Sciences, Dalian Medical University, Dalian, China; ^5^Institute of Biological Sciences, Jinzhou Medical University, Jinzhou, China

**Keywords:** PGE_2_, EP4, IgE class switching, STAT6, AKT, PPARγ, asthma

## Abstract

Increased serum IgE level is one of the features of allergic asthma. It is reported that IgE production can be enhanced by E-prostanoid 2 (EP2) receptor of prostaglandin E_2_ (PGE_2_); however, whether E-prostanoid 4 (EP4) receptor (encoded by *Ptger4*) has a unique or redundant role is still unclear. Here, we demonstrated the mice with B cell-specific deletion of the EP4 receptor (*Ptger4*^fl/fl^ *Mb1*^cre+/−^) showed their serum levels of IgE were markedly increased. A much more severe airway allergic inflammation was observed in the absence of EP4 signal using the OVA-induced asthma model. Mechanistic studies demonstrated that the transcription levels of AID, GLTε, and PSTε in EP4-deficient B cells were found to be significantly increased, implying an enhanced IgE class switch. In addition, we saw higher levels of phosphorylated STAT6, a vital factor for IgE class switch. Biochemical analyses indicated that inhibitory effect of EP4 signal on IgE depended on the activation of the PI3K-AKT pathway. Further downstream, PPARγ expression was up-regulated. Independent of its activity as a transcription factor, PPARγ here primarily functioned as an E3 ubiquitin-ligase, which bound the phosphorylated STAT6 to initiate its degradation. In support of PPARγ as a key mediator downstream of the EP4 signal, PPARγ agonist induced the down-regulation of phospho-STAT6, whereas its antagonist was able to rescue the EP4-mediated inhibition of STAT6 activation and IgE production. Thus, our findings highlight a role for the PGE_2_-EP4-AKT-PPARγ-STAT6 signaling in IgE response, highlighting the therapeutic potential of combined application of EP4 and PPARγ agonists in asthma.

## Introduction

Asthma is a chronic inflammatory disease characterized by airway hyperresponsiveness (AHR), chronic inflammation and tissue remodeling (1). The majority of cases of asthma are associated with IgE-mediated reactions reflecting atopy due to the critical role IgE plays in the development of asthma (2). The commitment of B cells to an IgE production is highly dependent on a unique type of intrachromosomal deletional recombination known as class switch recombination (CSR) (3). IgE synthesis is thought to occur through two main biosynthetic pathways, namely, by “direct” CSR from IgM in germinal center B-cells or by “sequential” switching from IgM to IgG1 and then from IgG1 to IgE, which may occur outside of germinal centers (4). The ε-switch CSR is induced by IL-4 and IL-13, which are secreted by activated Th2, ILC2, M2-macrophage and other cells (4). The binding of these cytokines to their receptors initiates a signaling cascade resulting in the phosphorylation of signal transducer and activator of transcription 6 (STAT6) and transfer to the nucleus. Then STAT6 binds to the ε germline gene promoter, initiating the transcription of “sterile” ε germline transcripts (εGLTs) (5) and later εGLTs as a target of activation-induced cytidine deaminase (AID) (6) ultimately lead to the recombination of the heavy chain between the Sμ and Sε regions. Usually, this ε-switch CSR is thought to be upregulated by IL-4 signal in combination with either anti-CD40 or lipopolysaccharide (LPS) (7, 8) *in vitro*. Activation of IL-4R and CD40 has synergistic effects that enhance both germline Cε transcription and AID transcription through NF-κB and STAT6 (9).

Many factors, including cytokines, B-cell surface receptors, transcription factors, and prostanoids, have been reported to regulate CSR in B cells (9). Among these, Prostaglandin E_2_ (PGE_2_) is thought to be a vital player. By binding to four different G-protein coupled receptors (EP1-4), PGE_2_ has pleiotropic effects in a wide array of tissues, ranging from cardiovascular, renal, respiratory, hematopoietic, and immune system tissues (10, 11). Up to now, an increasing number of studies suggest that PGE_2_ plays a role in the immunoregulation of cells ranging from ILCs, T cells to macrophages (11, 12). However, data from several studies on PGE_2_ on B cells seems controversial. An early example of research into the effect of PGE_2_ on B cells promotes the differentiation (isotype switching to IgE) of B cells in an LPS plus IL-4 system (13). Besides, pharmacological studies using agonists or antagonists suggest the impact of both EP2/EP4 receptors in PGE_2_-enhanced IgE production (13). However, another study by *Banchereau* group demonstrated that PGE_2_ can inhibit IL-4-induced IgG and IgE (14). Recently, a report showed that PGE_2_ promotes IgE production in an EP2-dependent manner in asthma (10). Nevertheless, what role EP4 receptor plays in IgE class switch and the definitive evidence regarding the cooperative or antagonistic roles of EP2 and EP4 as well as the mechanism are still missing.

PGE_2_ is a lipid mediator implicated in inflammatory diseases and in the regulation of lipolysis and adipocyte differentiation (15). According to several studies, in diet-induced obesity in rats, PGE_2_ inhibits liver lipolysis, β-oxidation, and very low density lipoprotein synthesis, further contributing to obesity (16). Moreover, the activation of EP4 signaling *in vitro* inhibits adipogenesis and adipocyte differentiation, thus restraining lipid accumulation in fat cells (17). PGE_2_ has also been reported to induce NR4A2 through EP4 to increase fatty acid oxidation (FAO) by inducing the expression of FAO genes (18). This evidence suggests that PGE_2_ plays an important role in the regulation of lipid metabolism. Meanwhile, the effects of PGE_2_ have been suggested to be linked to peroxisome proliferator-activated receptor γ (PPARγ) (19), which is a lipid-activated transcription factor essential for lipid metabolism (20). Currently, PPARγ is thought to be expressed in the lung and in a murine model of asthma, and treatment with a PPAR-γ agonist can inhibit the development of allergic inflammation, including pulmonary eosinophilia and airway AHR (21). However, whether PPARγ could influence on IgE production and whether PGE2 could regulate PPARγ in asthma was totally unclear. Based on the evidence above, we hypothesize that PGE_2_ may affect IgE class switching, and contribute to asthma development through its regulation on PPARγ.

## Materials and Methods

### Experimental Animals

WT C57BL/6 mice were brought from the animal breeding facility at Peking University Health Science Center (Beijing, China) under specific pathogen-free conditions. To generate mice lacking EP4 in B cells, mice with a conditional *loxP*-flanked *Ptger4* allele (*Ptger4*^fl/fl^) (22) were crossed with heterozygous *Mb1-cre* transgenic mice (23), both on C57BL/6 background. Mb1cre mouse were a gift from Prof. Haitao Shao, Institute of Biophysics, Chinese Academy of Sciences. All the mice used in the experiments were 8–12 weeks and were age- and sex-matched. All The experimental procedures on use and care of animals had been approved by the Ethics Committee of Peking University Health Science Center (Beijing, China).

### Chemicals and Reagents

PGE_2_ (14,010), PGE_1_-alcohol (13,020), and ONO-AE3-208 (14,522) were purchased from Cayman Chemical (Ann Arbor, MI, USA). OVA (A5503), LPS (L2880), and MG132 (M8699) were from Sigma (St. Louis, MO). MK2206 (S1078), Pioglitazone (S2590) and T0070907 (S2871) were from Selleck (Houston, TX, USA). The average 50% inhibition concentration (IC_50_) of T0070907 was determined as 1 nM.

### Airway Inflammation Models and Analytical Procedures

Mice were sensitized by intraperitoneal injections of 50 μg of OVA emulsified in 2 mg aluminum hydroxide (77,161; Thermo Fisher Scientific, Waltham, MA, USA) in a total volume of 200 μl on days 1, 8, 15, and challenged with an aerosol instillation of 1% OVA in PBS for 40 min on day 22, 24, 26. Control animals received phosphate-buffered saline (PBS) (P1020; Solarbio, Beijing, China) only. 24 h after the final challenge, the lung, serum and bronchoalveolar lavage fluid (BALF) were collected for further analysis. BALF was collected after flushing with 2 × 500 μl PBS and analyzed for the presence of inflammatory cells. For histology, lung tissues were fixed in 4% paraformaldehyde (P1110; Solarbio) and stained following standard protocols for HE and PAS.

In some models, WT mice were sensitized by OVA, then intranasally administered with different reagents in 20 μl, such as PGE_2_ (300 μg/kg), PGE_1_-alcohol (500 μg/kg), ONO-AE3-208 (1,000 μg/kg) or T0070907 (500 μg/kg), before an aerosol instillation of 1% OVA on day 22, 24, 26.

### Cell Sorting and Culture

Single-cell suspensions were obtained from the spleens of 6–10-wk-old mice and depleted of RBC by the ACK lysis buffer (R1010; Solarbio). Purified total splenic B cells were obtained via magnetic separation using CD19 beads (130-097-144; Miltenyi Biotec) as described. Purity was determined to be >97% by FACS analysis. Purified B cells were cultured at 1 × 10^6^ cells/ml in Opti-MEM (31,985,088; Gibco, Carlsbad, Calif) supplemented with 10% fetal calf serum (FCS). To induce IgE production, cells were stimulated with LPS (20 μg/ml) or anti-CD40 (1 μg/ml, Clone HM40-3, 553,721; BD Pharmagin, San Jose, Calif) plus IL-4 (50 ng/ml, 214-14; PEPROTECH, Rocky Hill, NJ) for 3–7 days in the presence or absence of PGE_2_.

### Enzyme-Linked Immunosorbent Assay (ELISA)

Total IgE in the serum and culture supernatant was measured using OptEIA™ Mouse IgE ELISA Set (555,248; BD Biosciences, San Jose, Calif) according to the manufacturer's instruction as described. To measure serum levels of OVA-specific IgE, serially diluted sera was added into 96-well plates pre-coated with anti-IgE (2 μg/ml), followed by incubation with biotinylated OVA (1.25 μg/ml). The bound biotinylated OVA was detected with horseradish peroxidase (HRP)-conjugated streptavidin using tetramethylbenzidine as substrate. The absorbance was read at 450/490 nm.

### Flow Cytometry

Single cells isolated from the spleen were stained for 30 min with the appropriate fluorescence-conjugated antibodies and washed, then were resuspended with PBS. Flow cytometric analysis was performed on BD FACSCanto Plus using Kaluza software. Anti-IL4Rα (144,803), Annexin V, 7-AAD, B220 were from Biolegend. Anti-EP4 Receptor (C-Term, 16,625) was from Cayman chemical.

### Confocal Fluorescence Microscopy

B cells were fixed with 4% paraformaldehyde overnight and permeabilized with 0.1% TritonX-100 (T8200; Solarbio) for 10 min. The fixed cells were blocked with blocking buffer (SW3015; Solarbio) at 37°C for 1 h. Then cells were stained with the primary antibody anti-STAT6 and anti-PPARγ overnight at 4°C. After three washes with PBS, the cells were incubated for another 1 h with secondary Alexa Fluor 555-conjugated anti-rabbit IgG or Alexa Fluor 488-conjugated anti-mouse IgG antibody (Zhongshan Golden Bridge Biotechnology, Beijing, China). The DNA was stained with 4′-6′-diamidino-2-phenylindole (DAPI, D9542; Sigma) at room temperature for 5 min. Specimens were captured with a confocal microscope.

Multiplex immunofluorescence staining was obtained using PANO 4-plex IHC kit (Panovue, Beijing, China, 0,001,100,100). Different primary antibodies were sequentially applied, followed by HRP-conjugated secondary antibody incubation and tyramide signal amplification. The slides were heat-treated with Antigen retrieval solution (Citric acid solution, pH6.0/pH9.0) at 100°C after each TSA operation. Nuclei were stained with DAPI after all the antigens had been labeled. All images were collected with a confocal microscope. Antibodies were used as follow: anti-Rab5 (1:200 dilution; Cell signaling, 46449); Anti-EP4 Receptor (1:100 dilution; Cayman chemical, 16,625); Anti-EP2 Receptor (1:100 dilution; Cayman chemical, 10,477); anti-Phospho-STAT6 (Tyr641) (1:200 dilution; Cell signaling, 56,554); anti-STAT6 (1:200; Proteintech, Rosemont, IL, 66717-1-Ig); anti-PPARγ (1:200 dilution; Cell signaling, 2,443); anti-ubiquitin (1:200 dilution; Cell signaling, 3,936).

### Quantitative RT-PCR

Total RNA from cultured B cells was isolated with Trizol Reagent (15,596,018; Invitrogen, Life Technologies). cDNA was synthesized using the AMV cDNA Reverse Transcription Kit (A3500; Promega, Madison, WI). For quantitative PCR, SYBR Green Supermix (11143ES50; YEASEN, Beijing, China) was used according to the manufacturer's instructions. The amplification was performed on an iCycler (Bio-Rad Laboratories, Hercules, CA). β-actin was used as an internal control.

The primers used are listed in Table 1.

**Table 1:** Primers used for real-time PCR.

**Gene name**	**Sequence(5′ -> 3′)**
Aicda	F: TGCTACGTGGTGAAGAGGAG
	R: TCCCAGTCTGAGATGTAGCG
GLTε	F: GCACAGGGGGCAGAAGAT
	R: CCAGGGTCATGGAAGCAGTG
PSTε	F: TTGGACTACTGGGGTCAAGG
	R: CAGTGCCTTTACAGGGCTTC
Hsp90aa1	F: TGTTGCGGTACTACACATCTGC
	R: GTCCTTGGTCTCACCTGTGATA
Nos3	F: GGCTGGGTTTAGGGCTGTG
	R: CTGAGGGTGTCGTAGGTGATG
Mtor	F: GGTGCTGACCGAAATGAGGG
	R: GCGTGGACCCATCTCTCAC
Pparg	F: TGTGGGGATAAAGCATCAGGC
	R: CCGGCAGTTAAGATCACACCTAT
β-actin	F: TATGGAATCCTGTGGCATC
	R: GTGTTGGCATAGAGGTCTT

### Western Blotting

B cells cultured under various conditions were harvested and cell lysates were prepared in lysis buffer. The lysate was resolved on a 10% reducing SDS-polyacrylamide gels and transferred to a polyvinylidene difluoride membrane. After blocking with Tris-buffered saline (pH 7.4) containing 5% dried skimmed milk, the membrane was incubated with antibodies, followed by probing with HRP-conjugated anti-rabbit or -mouse antibody (Sigma). The bands were detected by chemiluminescence with ECL detection reagents (Life Technologies). Antibody to Phospho-STAT6 (Tyr641, 56,554), anti-STAT6 (5,397), anti-p-PKA C(4,781), anti-PKA C-α (5,842), anti-p-CREB (9,198), anti-CREB (9,197), PI3 Kinase p110 δ 34,050, anti-p-Akt (Ser473, 4,060), anti-Akt (4,685), anti-p-FoxO1 (Ser256, 9,461), anti-FoxO1 (2,880), anti-p-FoxO3a (Ser253, 13,129), anti-FoxO3a (12,829), anti-PPARγ (2,443) were from Cell Signaling Technology (Danvers, MA, USA). Anti-ubiquitin (PTM-1106) was from PTM BIO (Beijing, China). Anti-β-Actin (66009-1-Ig) was from Proteintech.

### Immunoprecipitation (IP)

5 × 10^7^ B cells were lysed with 500 μl IP buffer (50 mM Tris-HCl at pH 7.5, 150 mM NaCl, 1 mM EDTA and 0.5% NP-40) containing Phosphatase protease inhibitors (PPC1010; Sigma) for 1 h at 4°C, and then sonicated for 36 s on ice. After centrifugation at 13,000 rpm for 10 min, the protein concentrations were measured, and equal amounts of the lysate were used for IP. IP was performed with appropriate antibodies overnight at 4°C, followed by adding protein A-Sepharose (17-0780-01; GE Healthcare, Waukesha, Wis) for addition 4 h. Thereafter, the precipitants were washed with IP buffer, eluted with sample buffer containing 1% SDS, separated by SDS-PAGE, and immunoblotted with specific antibodies and secondary anti-mouse or anti-rabbit antibodies conjugated to horseradish peroxidase (Sigma).

### *In vivo* Ubiquitination Assay

1 × 10^7^ B cells were treated with 20 μM MG132 for 10 h before 30 min stimulation of anti-CD40+ IL4, then lysed with denaturing buffer (1% sodium dodecyl sulfate, 50 mM Tris at pH 7.5, 150 mM NaCl, 1 mM EDTA) to disrupt protein–protein interactions. Next, the lysates were diluted 10 times with IP buffer (50 mM Tris-HCl at pH 7.5, 150 mM NaCl, 1 mM EDTA and 0.5% NP-40) and subjected to IP with anti-p-STAT6 antibody overnight at 4°C. Bound proteins were released from beads by boiling in sodium dodecyl sulfate–polyacrylamide gel electrophoresis sample buffer followed by immunoblotting with anti-ubiquitin antibody.

### Protein–Protein Interaction Network (PPI)

The protein–protein interaction network is constructed by STRING (https://string-db.org/). Briefly, the differentially expressed genes were uploaded and then analyzed by the STRING Interactome PPI database (Version 11.0). Then, by the topology and module analysis, the key nods and modules are identified.

### Ethics Statement

This study was carried out in accordance with the recommendations of Ethics Committee of Peking University Health Science Center. The protocol (No. LA2018106) was approved by the Ethics Committee of Peking University Health Science Center.

### Statistical Analysis

Statistical differences were determined by one-way ANOVA with Tukey's multiple comparisons test or by unpaired two-tailed Student's *t*-test using GraphPad Prism software (GraphPad Software, San Diego CA). Data are presented as mean ± SD unless otherwise specified. ^*^p < 0.05, ^**^p < 0.01, and ^***^p < 0.001, ns, not significant.

## Results

### EP4 Deficiency Leads to Elevated IgE and Exaggerated Asthmatic Responses to OVA

**Figure 1:**
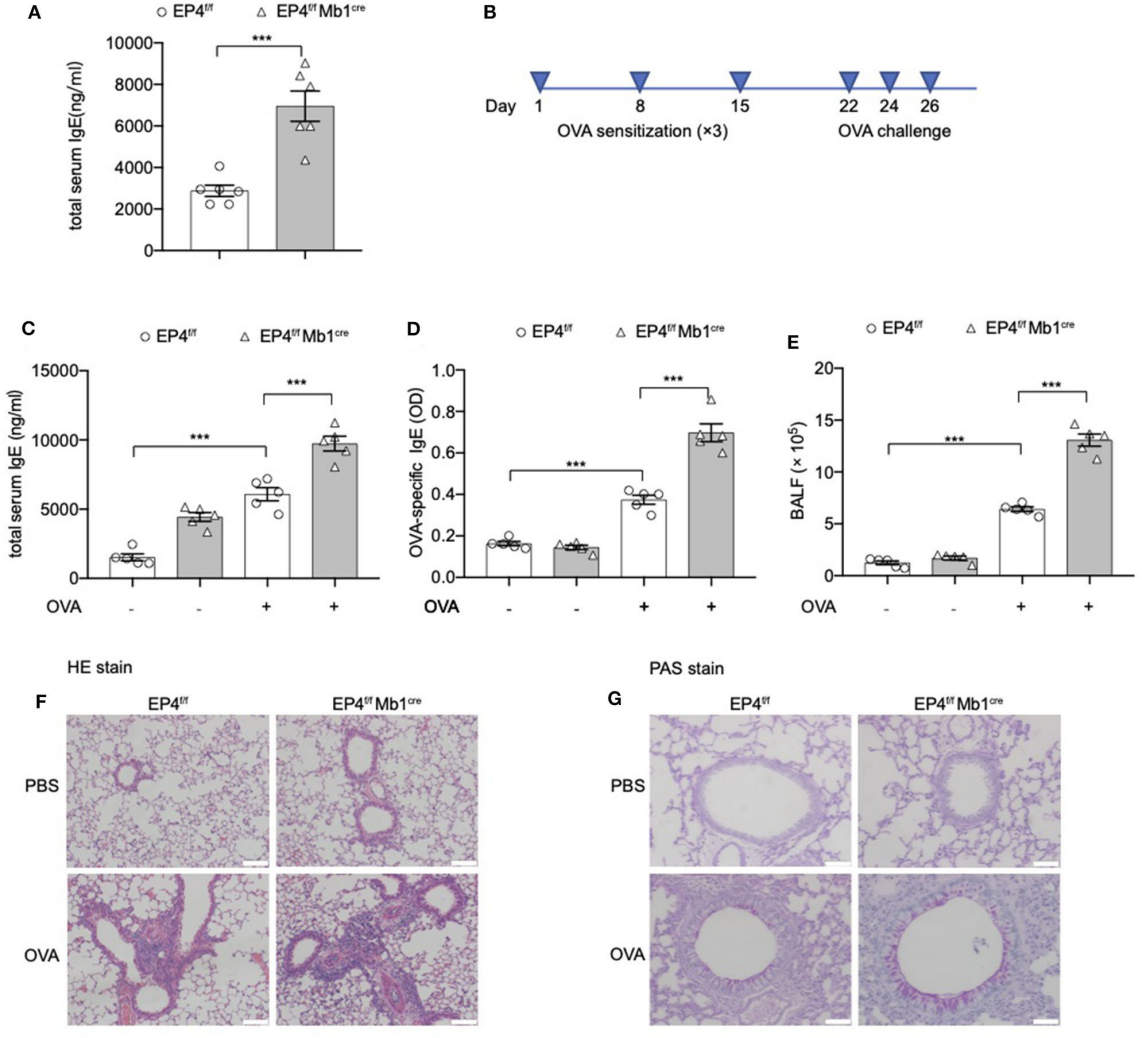
Exaggerated asthmatic responses in EP4-deficiency mice. **(A)** Serum level of total IgE of *Ptger4*^fl/fl^ (EP4^f/f^) and *Ptger4*^fl/fl^*Mb1*^cre+/−^ (EP4^f/f^ Mb1^cre^) were detected by ELISA (*n* = 6). **(B)** EP4^f/f^ and EP4^f/f^ Mb1^cre^ were immunized with OVA following a protocol as described in the methods. **(C,D)** Serum levels of total **(C)** and OVA-specific **(D)** IgE were determined by ELISA 24 h after the last challenge (*n* = 5). **(E)** Total cell number in the BALF (*n* = 5). **(F,G)** Representative images showing hemotoxylin and eosin (HE) **(F)** and Periodic Acid-Schiff (PAS) **(G)** staining of the lung tissue. Scale bar equals 100 μm **(F)** or 50 μm **(G)**. Data are presented as mean ± SD, representing one of three independent experiments. Statistical differences were determined by one-way analysis of variance (ANOVA) with Tukey's multiple comparisons test **(C–E)** or by unpaired two-tailed Student's *t*-test **(A)**. ***P < 0.001.

Data from several studies suggest that prostaglandin E_2_ (PGE_2_) plays a role in IgE production, but conflicting data have been reported (10, 14, 24–27). Previously, our lab also showed that, in EP2-deficient mice, serum IgE is present at lower levels than in wild-type littermates (WT) (10). However, it remains to be determined whether EP4 (encoded by *Ptger4*) has a unique or redundant role. In the present study, we constructed *Ptger4*^fl/fl^ *Mb1*^cre+/−^ mice (EP4^f/f^ Mb1^cre^), which can specifically delete *Ptger4* in the early stage of B cells, to examine how PGE_2_-EP4 signal modulates IgE production. From our data, EP4^f/f^ Mb1^cre^ mice exhibited an elevated level of serum IgE production than *Ptger4*^fl/fl^ mice (EP4^f/f^) in the physiological state (Figure 1A). Of note, this result is contradictory to the reduced serum IgE in EP2-deficient mice, suggesting that EP2 and EP4 signal has differential influence on IgE production. We then examined other receptors' expression and the development and early activation of EP4^f/f^ Mb1^cre^ B cells. In EP4^f/f^ Mb1^cre^ mice, the expression of other PGE_2_ receptors (EP1, EP2, EP3) was only had slight change with the lack of EP4 (data unpublished). Also, EP4^f/f^ Mb1^cre^ mice had fairly normal development of B cells in the BM, spleen and lymph nodes (Figures S1A–D). In addition, EP4^f/f^ Mb1^cre^ mice also showed no big change of co-stimulatory molecules, including CD80, CD86, IA/IE (Figure S1E), implying the PGE_2_-EP4 signal has no effect on the activation status of peripheral B cells in the resting state. Thus, these data suggested that the alteration of IgE may be due to the later phase of B cells, such as the differentiation of plasma cells or CSR.

Then we explored the pathological consequence of EP4 deletion using the OVA-induced asthma model (Figure 1B). As expected, a much more severe airway allergic inflammation was observed in the absence of EP4 signal. EP4^f/f^ Mb1^cre^ mice showed nearly 2-fold increase in the total serum IgE concentration compared with EP4^f/f^ mice after OVA immunization (Figure 1C). OVA-specific IgE data had the similar result (Figure 1D). The bronchoalveolar lavage fluid (BALF) (Figure 1E) demonstrated that a larger number of inflammatory cells infiltrated in the airway of OVA-immunized EP4^f/f^ Mb1^cre^ mice. Moreover, hematoxylin and eosin (HE) (Figure 1F) as well as periodic acid-Schiff (PAS) staining (Figure 1G) of the lung tissue showed more intensive peribranchial lymphoid infiltration and increased mucus production. Therefore, our data suggested that allergic inflammation in EP4^f/f^ Mb1^cre^ mice was much more pronounced than that in EP4^f/f^ mice and that PGE_2_ may play an inhibitory role in regulating the development of IgE secretion through the EP4 receptor.

### EP4-Mediated PGE_2_ Signaling Inhibits IgE Production Induced by Anti-CD40 Plus IL4

**Figure 2:**
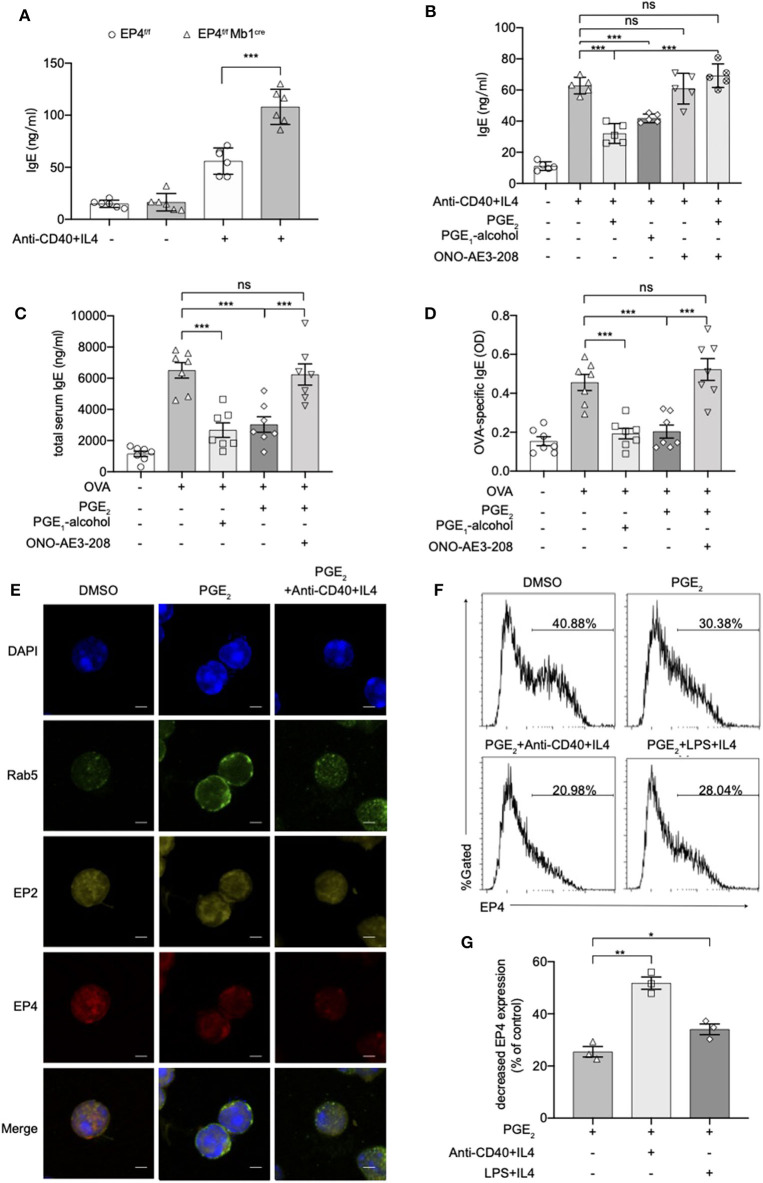
Anti-CD40 + IL4 amplifies the effect of PGE_2_ on EP4. **(A)** Splenic B cells from EP4^f/f^ or EP4^f/f^ Mb1^cre^ were stimulated with anti-CD40 (1 μg/ml) + IL4 (50 ng/ml). IgE concentrations in the culture supernatant at day 7 were measured by ELISA (*n* = 6). **(B)** ELISA of IgE levels from WT B cells treated with anti-CD40+ IL4 with or without concomitant administration of PGE_2_ (10 nM), PGE_1_-alcohol (1 μM), or ONO-AE3-208 (10 μM) for 7 days (*n* = 5). **(C,D)** Mice were treated with OVA with or without concomitant administration of PGE_2_ (300 μg/kg), PGE_1_-alcohol (500 μg/kg), or ONO-AE3-208 (1,000 μg/kg) following a protocol as described in the methods. Airway inflammatory responses were analyzed 24 h after the last challenge. Serum levels of total **(C)** and OVA-specific **(D)** IgE were determined by ELISA (*n* = 7). Each symbol represents an individual mouse. **(E)** Confocal microscopy of the expression of Rab5, EP2, and EP4 immunostaining in the indicated colors in WT B cells under different stimulations for 15 min. Scale bars, 2.5 μm. Data in **(E)** are representative data of three independent experiments. **(F,G)** Surface expression of EP4 was determined by flow cytometry at 15 min. Representative histogram **(F)** and the percentage of decreased EP4 expression **(G)** are shown (*n* = 3). Control means unstimulated B cells. Data in **(C–F)** are representing one of three independent experiments. Data in **(A,B,G)** are pooled from three independent experiments. Statistical differences were determined by one-way ANOVA with Tukey's multiple comparisons test **(A–G)**. Data are presented as mean ± SD. ^*^p < 0.05, ^**^p < 0.01, and ^***^p < 0.001, ns, not significant.

To verify whether these exaggerated asthmatic responses in EP4^f/f^ Mb1^cre^ mice were due to the overwhelming IgE production, we purified splenic B cells from EP4^f/f^ Mb1^cre^ and EP4^f/f^ mice and cultured them under anti-CD40+IL-4 to examine their IgE secretion. As Figure 2A shows, compared with EP4^f/f^ B cells, EP4-deficient B cells did double the IgE level after 5–7 days' culture. To confirm the importance of EP4 signal in anti-CD40 plus IL-4 induced IgE production, we cultured B cells with an EP4 selective agonist, PGE_1_-alcohol (28), and an EP4 antagonist, ONO-AE3-208 (AE3-208) (29). As shown in Figure 2B, the increase in the anti-CD40 and IL-4-driven production of IgE was effectively blocked by the addition of PGE_2_ and this inhibitory effect was mimicked by PGE_1_-alcohol. In contrast, EP4 antagonist AE3-208 treatment in WT can markedly rescue IgE level. Furthermore, although the antagonist's effect on endogenous PGE_2_ was slight, the exogenous PGE_2_' inhibition was abrogated by AE3-208. In OVA-challenged model, which is widely accepted as a Th2-type asthma model (30), EP4 agonist PGE_1_-alcohol displayed similar potency as PGE_2_ in inhibiting OVA-induced airway inflammation based on all parameters examined, including IgE concentration, the exudation of inflammatory cells, and lung pathology (Figures 2C,D, Figure S2). Consistently, the addition of the EP4 antagonist AE3-208 can effectively rescue the inhibitory effect of PGE_2_. Interestingly, PGE_2_ signal through EP4 receptor seemed have a higher sensitivity to anti-CD40 stimulation. Figures 2E–G illustrated that the exposure to PGE_2_ induced a decrease in the expression of both the EP4 and EP2 receptors, possibly indicating receptor endocytosis, however, the exposure to anti-CD40+IL4+PGE_2_ exhibited a greater decrease in the expression of the EP4 receptor, in spite of no further changes of EP2 receptor expression. Notably, this decrease on EP4 was anti-CD40 selective preference since LPS+IL-4+PGE2 didn't have similar synergic effect (Figures 2F,G). In summary, these results indicate that EP4-mediated signaling affect the development of Th2-type asthma and this receptor is the key media in the regulation of CD40-induced IgE production.

### PGE_2_-EP4 Signal Inhibits IgE Class Switching

**Figure 3:**
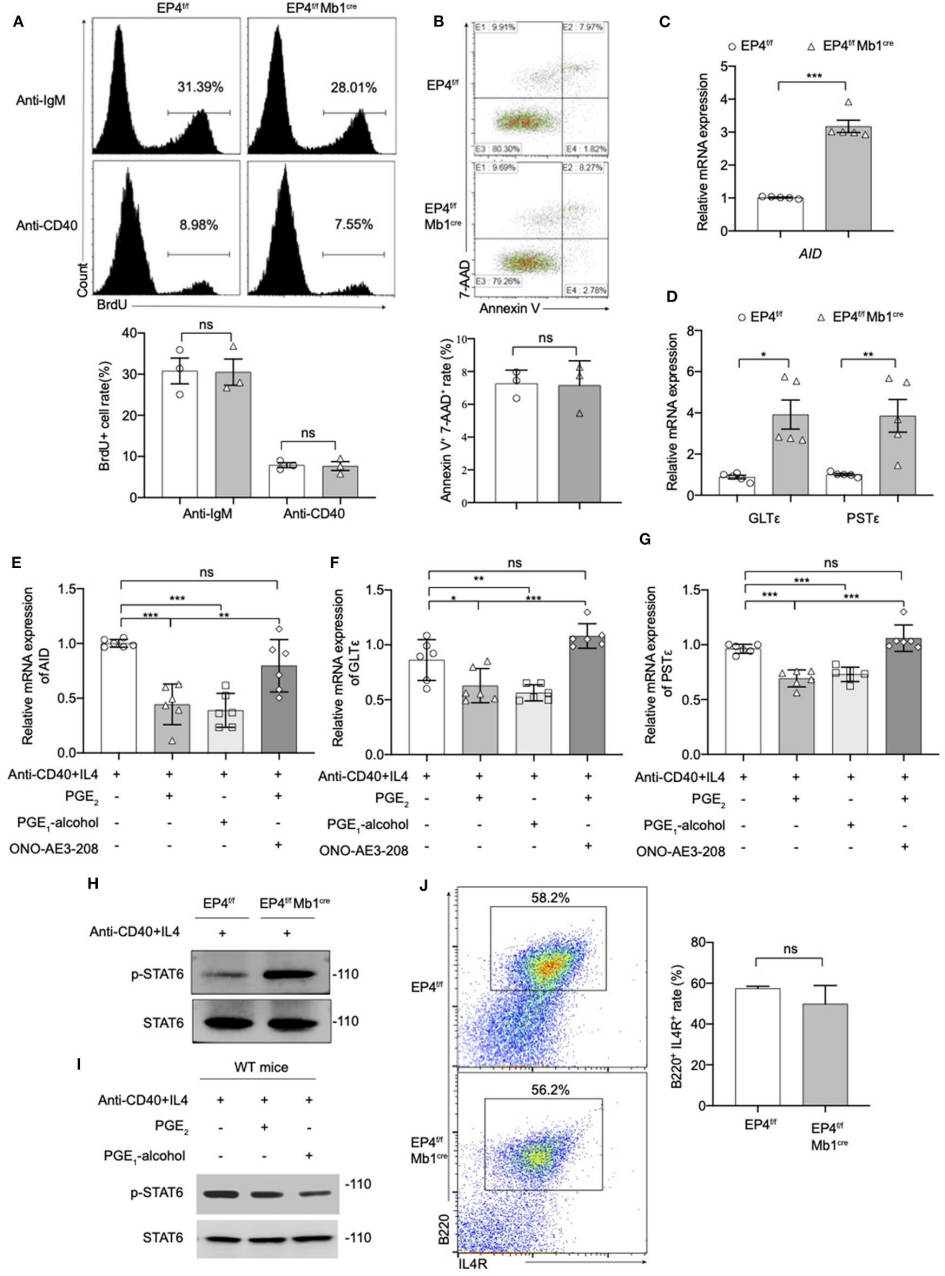
Modulation of the pSTAT6 signaling by EP4-mediated signal. **(A)** Proliferation assays were identified by Brdu incorporation with the stimulation of anti-IgM or anti-CD40. Representative histogram and the percentage of BrdU^+^ B cells are shown (*n* = 3). **(B)** Apoptosis assays were determined by flow cytometry after the stimulation of anti-IgM 24 h. Representative histogram and the percentage of Annexin V^+^ 7-AAD^+^ B cells are shown (*n* = 3). **(C,D)** RNA was prepared from day 3 cultures of EP4^f/f^ and EP4^f/f^ Mb1^cre^ B cells in the presence of CD40+IL4 and examined for AID **(C)**, GLTε and PSTε **(D)** mRNA expression by quantitative PCR (*n* = 5). **(E,F,G)** RT-qPCR analysis of AID **(E)**, GLTε **(F)**, and PSTε **(G)** on WT B cells from day 3 cultures with indicated groups (*n* = 6). **(H,I)** Splenic B cells from EP4^f/f^ or EP4^f/f^ Mb1^cre^ mice were stimulated with anti-CD40+ IL-4 in the presence or absence of PGE_2_ and PGE_1_-alcohol. Cells were harvested at 30 min after stimulation. Phosphorylated and total STAT6 was detected by Western blotting. Representative blots are shown of three independent experiments. **(J)** Expression of IL4R was determined by flow cytometry. Representative histogram and the percentage of B220^+^IL4R^+^ B cells are shown. Data in **(A,B,H,J)** are representative data of three independent experiments. Data are pooled from two or three independent experiments. Statistical differences were determined by one-way **(E,F,G)** ANOVA with Tukey's multiple comparisons test or by unpaired two-tailed Student's *t*-test **(A–D,J)**. Data are presented as mean ± SD. ^*^p < 0.05, ^**^p < 0.01, and ^***^p < 0.001, ns, not significant.

We next explored the cellular and molecular mechanisms behind the regulatory role of EP4 using B cell cultures under IgE-inducing conditions. This study first examined the proliferation of B cells in EP4^f/f^ Mb1^cre^ stimulated for 72 h under anti-IgM or anti-CD40 conditions. BrdU staining results showed there was no significant difference from the proportion of proliferating cells between the EP4^f/f^ Mb1^cre^ and EP4^f/f^ B cells. This suggested that the absence of EP4 didn't affect the proliferation of B cells (Figure 3A).

Next, we investigated the effect of EP4 deletion on B cell apoptosis. B cells were stimulated with anti-IgM for 24 h, and then tested using AnnexinV-7-AAD combined staining. By counting the AnnexinV ^+^ 7-AAD ^+^ cells, it was found there was no significant difference in the rate of apoptotic cells in EP4^f/f^ Mb1^cre^ mice compared with the control group (Figure 3B). The above results suggested that the absence of EP4 signal didn't affect the activation process of B cells after stimulation. Combined with previous research suggesting that PGE_2_ can affect the class switching of IgE (10), we turned to examine whether the class switch recombination (CSR) of IgE is affected by the absence of EP4.

It is reported that the key molecules in the process of IgE CSR include activation-induced cytidine deaminase (AID), germ-line transcripts ε (GLTε), and Post-switch transcripts ε (PSTε) (9, 31–34). Therefore, this study examined the AID, GLTε and PSTε levels in B cells cultured for 3 days as described above. Similar to what was observed for IgE expression, the results showed that GLTε, PSTε, and AID expression in B cells of EP4^f/f^ Mb1^cre^ mice were increased nearly 3-4 times compared to control mice (Figures 3C,D). Furthermore, we used EP4 agonist PGE_1_-alcohol to simulate EP4 signal on WT B cells. The results showed that the EP4 agonist PGE_1_-alcohol had the similar effect of PGE_2_ on the expression of these molecules, and the EP4 antagonist ONO-AE3-208 could rescue their mRNA level from the suppression of exogenous PGE_2_ (Figures 3E–G).

STAT6 is known as a key mediator of the induction of IgE production by IL-4 (35).

In addition, STAT6 is particularly involved in class switching to IgE through the generation of germline transcripts of Cε (GLTε), as well as the expression of activation-induced cytidine deaminase (AID) (36). To determine whether the downstream of IL-4 was affected by EP4-mediated PGE_2_ signal, we next examined the phosphorylation of STAT6 (p-STAT6). Figure 3H illustrates that in EP4^f/f^ Mb1^cre^ mice phospho-STAT6 was significantly upregulated by anti-CD40 and IL4 within 30 min (Figure 3H). In contrast, PGE_2_ as well as EP4 agonist treatment in WT can markedly down-regulate phospho-STAT6 (Figure 3I). To check whether the decrease in pSTAT6 is partly due to changes in the expression of IL-4Rs, we measured the expression of IL-4R using flow cytometry. The results showed its level in EP4^f/f^ Mb1^cre^ might have a slight decrease but not significant compared with control mice (Figure 3J). Collectively, the impact of EP4 on IgE production seems to be largely attributable to the alteration of phospho-STAT6 and its regulation of consequent CSR.

### PGE_2_-EP4 Signaling Promotes the Phosphorylation of Akt to Inhibit Phospho-STAT6 and IgE Production

**Figure 4:**
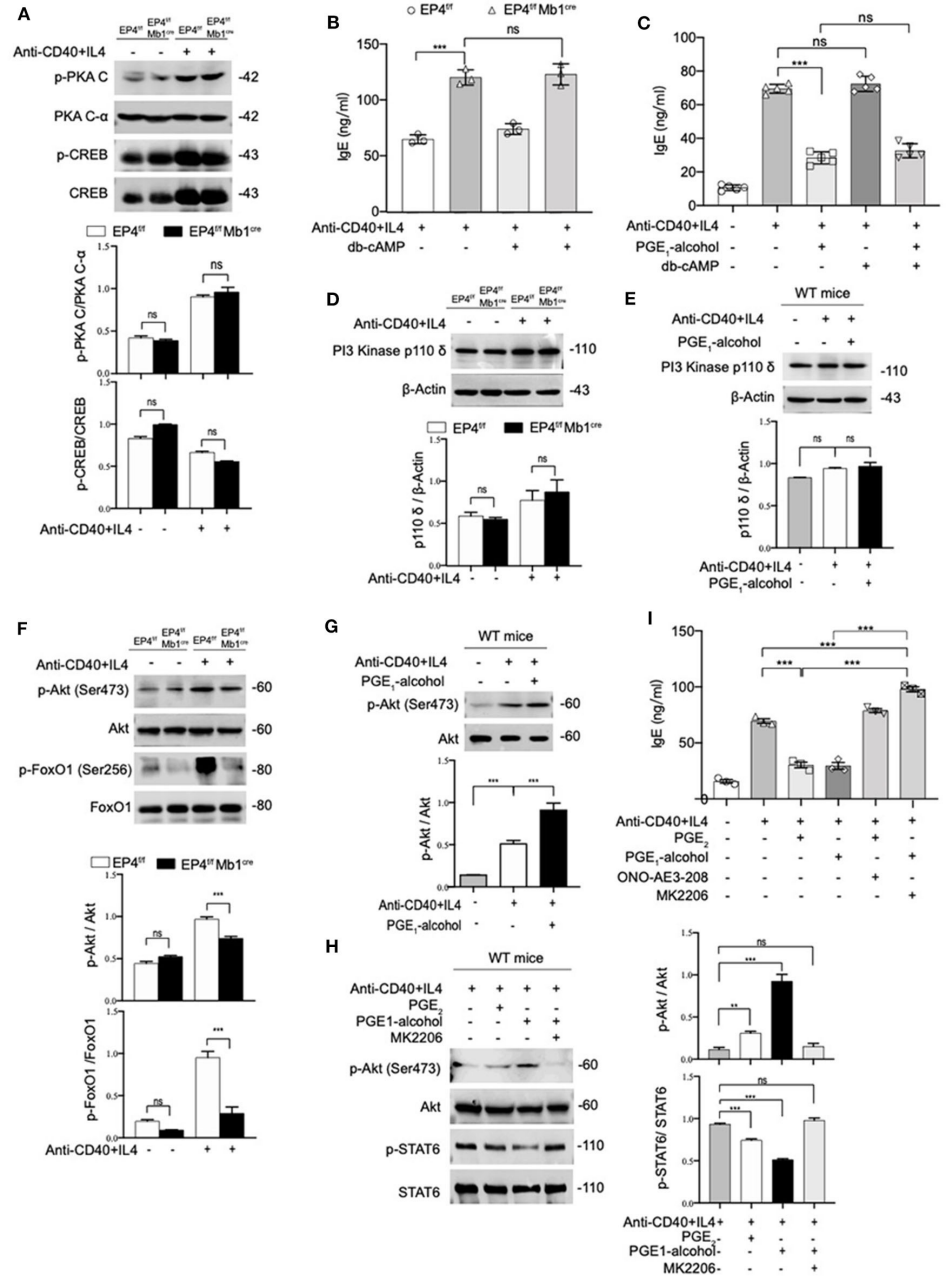
PGE_2_-EP4 Signaling Promotes the Phosphorylation of Akt to Regulate pSTAT6. **(A)** Immunoblot analysis of p-PKA C, PKA C-α, p-CREB, CREB treated with anti-CD40+ IL-4 for 30 min. **(B,C)** B cells were stimulated with anti-CD40+ IL-4 in the presence or absence of dibutyryl cAMP (db-cAMP) (4 μM) and the cultures were analyzed on day 7. **(D)** PI3 Kinase p110 δ in EP4^f/f^ and EP4^f/f^ Mb1^cre^ B cells treated with anti-CD40+ IL-4 for 30 min. **(E)** Immunoblot analysis of PI3 Kinase p110 δ in WT B cells treated with anti-CD40+ IL-4 in the presence or absence of PGE_1_-alcohol (1 μM) for 30 min. **(F)** Immunoblot analysis of p-Akt (Ser473), Akt, p-FoxO1 (Ser256), and FoxO1in EP4^f/f^ and EP4^f/f^ Mb1^cre^ B cells treated with anti-CD40+ IL-4 for 30 min. **(G)** Immunoblot analysis of p-Akt (Ser473) and Akt in WT B cells treated with anti-CD40+ IL-4 in the presence or absence of PGE_1_-alcohol (1 μM) for 30 min. **(H)** Immunoblot analysis of p-STAT6, STAT6, p-Akt (Ser473), and Akt in WT B cells treated with anti-CD40+ IL-4 in the presence or absence of PGE_2_ (10 nM), PGE_1_-alcohol (1 μM), and MK2206 (0.5 μM) for 30 min. **(I)** ELISA of IgE levels from WT B cells treated with anti-CD40+ IL4 with or without concomitant administration of PGE_2_ (10 nM), PGE_1_-alcohol (1 μM), or ONO-AE3-208 (10 μM), MK2206 (0.5 μM) for 7 days (*n* = 3). Data in **(A,D,E,F,G,H)** are representative data of three independent experiments. Data are pooled from three independent experiments. Statistical differences were determined by one-way ANOVA with Tukey's multiple comparisons test. Data are presented as mean ± SD. ^**^p < 0.01 and ^***^p < 0.001, ns, not significant.

Various studies have found that PGE_2_ stimulation of the EP4 receptor was through two main pathways (37). One is that cAMP-dependent protein kinase (PKA) and CRE-binding protein (CREB) pathway and this path is shared by EP2 and EP4. The other is PI3K-Akt signaling, which is an exclusive pathway of EP4 (38, 39). Therefore, we detected both PKA/CREB and PI3K/Akt signaling under anti-CD40+IL-4 stimulation. As shown in Figure 4A, phospho-CREB and phospho-PKA C were not significantly changed in EP4^f/f^ Mb1^cre^ mice (Figure 4A). To further confirm the role of the cAMP pathway in the production of IgE, we introduced Dibutyryl cAMP (db-cAMP), a cell-permeable cAMP analog. The ELISA results showed that in the anti-CD40 + IL-4 system, the IgE level of EP4^f/f^ Mb1^cre^ remained unaltered despite of the addition of db-cAMP (Figure 4B). After adding db-cAMP to WT B cells, there was no change in IgE levels compared with the anti-CD40 + IL-4 treatment alone. Adding db-cAMP to anti-CD40 + IL-4 and PGE1-alcohol treatment groups, IgE levels have not been significantly affected (Figure 4C). Therefore, the regulation of cAMP does not affect the production of IgE, and the inhibitory effect of PGE_2_ on IgE production may not depend on the cAMP/PKA/CREB pathway.

On the other hand, we tested the expression levels of related molecules in EP4 unique pathway, the PI3K-Akt pathway (40). Western blot analysis showed no significant changes in the PI3K p110 δ subunit (40) of B cells in EP4^f/f^ Mb1^cre^ and control mice (Figure 4D). Likewise, the addition of EP4 agonist PGE_1_-alcohol in WT B cells did not change its expression level (Figure 4E). However, the immunoblot analysis of EP4^f/f^ Mb1^cre^ B cells showed significant deficiency of phosphorylated Akt and strong deficiency of its principal target FoxO1 (41) after 30 min post-stimulation by anti-CD40 plus IL-4 (Figure 4F). In contrast, the addition of the EP4 agonist PGE_1_-alcohol strengthened the activation of Akt (Figure 4G). Therefore, we preliminarily speculate that the inhibitory effect of EP4 signaling depends on the activation of the AKT pathway.

To fully address the role of Akt in the EP4-mediated inhibition of IgE differentiation, we used a highly selective allosteric inhibitor of Akt, MK2206. MK-2206 has been reported to suppress the activity of AKT by decreasing the phosphorylation of AKT S473 (42). In our system, the addition of Akt inhibitor MK2206 can significantly inhibit the serine 473 phosphorylation of Akt (Figure 4H). It is worth noting that in the presence of MK2206, the inhibitory effect of EP4 agonist PGE1-alcohol on phospho-STAT6 can be completely rescued (Figure 4H). This indicates that Akt activation has a very important role in regulating phospho-STAT6 levels. We then continued to observe the effect of the Akt inhibitor MK2206 on IgE. We cultured WT B cells under different conditions, and collected the supernatant to detect the secreted IgE levels. Its rescue of PGE_2_-EP4 signaling was also observed in IgE titers. Along with enhancing the phosphorylation of STAT6, MK2206 treatment (column 6), compared with PGE_1_-alcohol treatment alone (column 4), led to an ~3-fold increase in IgE levels (Figure 4I). Briefly, these data indicate that PGE_2_-EP4 signaling primarily functions through the Akt pathway instead of the cAMP pathway in B cells, inhibiting the activation of STAT6 and then suppress the production of IgE.

### PGE_2_-EP4 Signaling Up-Regulates PPARγ Expression by Akt Activation

After clarifying that EP4 inhibited IgE by activating Akt, we further explored the mechanism by which EP4-Akt signaling affects phospho-STAT6. Previous studies have shown that the selective EP4 agonist L-902,688 can treat idiopathic pulmonary hypertension by activating peroxisome proliferator-activated receptor γ, PPARγ (43).

**Figure 5:**
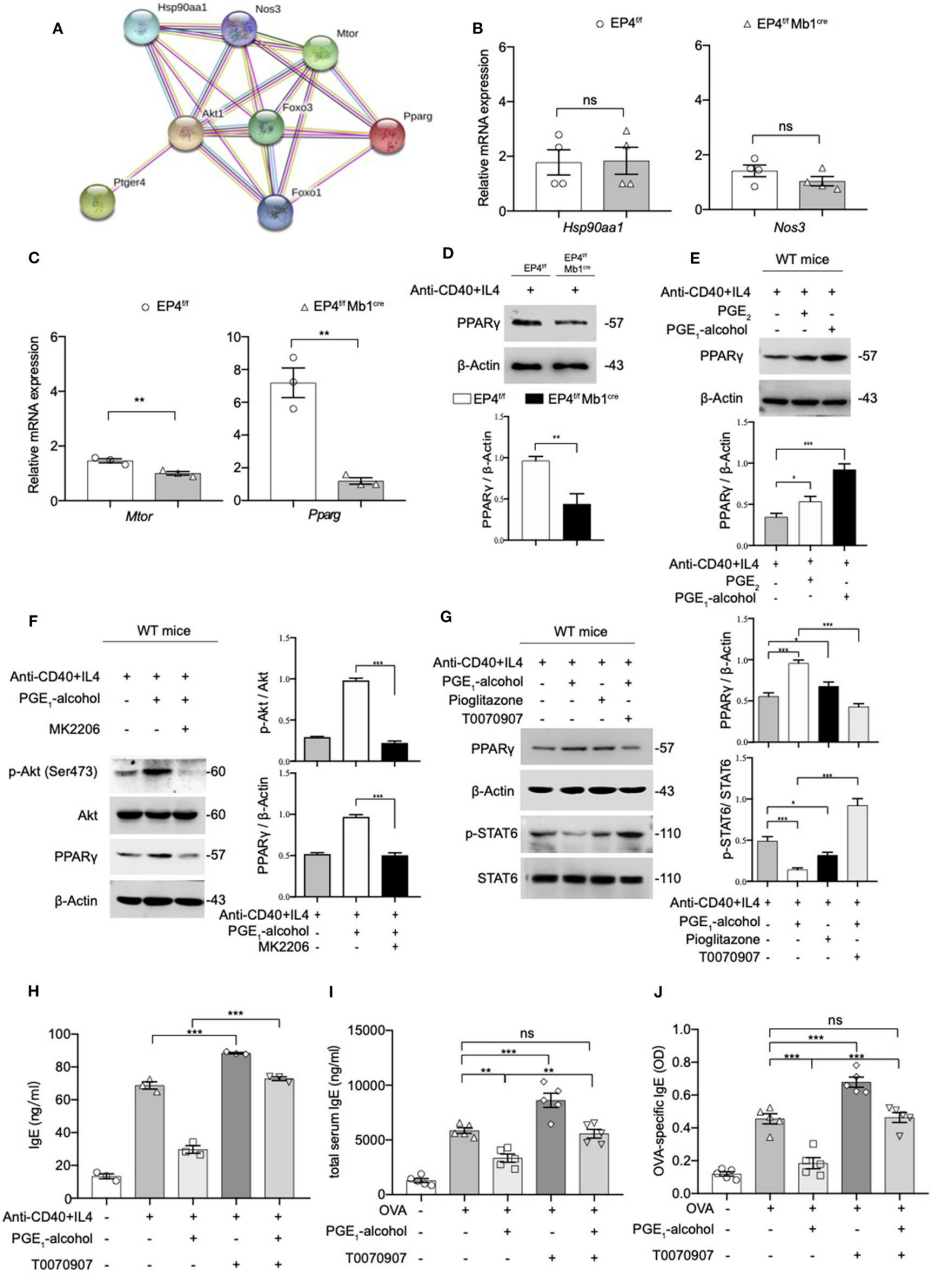
The Phosphorylation of STAT6 is Mediated by PPARγ. **(A)** Protein–protein interaction network (PPI) of Ptger4, Akt, FoxO3, and PPARγ, PPI enrichment *p*-value: 0.000834. **(B,C)** RT-qPCR analysis of the *Hsp90aa1, Nos3, Mtor*, and *Pparg* gene expression in EP4^f/f^ and EP4^f/f^ Mb1^cre^ B cells for 3 days [*n* = 4**(B)**, 3**(C)**]. (mean ± SEM) **(D)** Immunoblot analysis of the *Pparg* gene expression in EP4^f/f^ and EP4^f/f^ Mb1^cre^ B cells for 3 days. **(E)** Immunoblot analysis of PPARγ in WT B cells treated with anti-CD40+ IL-4 in the presence or absence of PGE_2_ (10 nM) and PGE_1_-alcohol (1 μM) for 30 min. **(F,G)** Immunoblot analysis of PPARγ, p-STAT6, STAT6, p-Akt (Ser473), and Akt in WT B cells treated with anti-CD40+ IL-4 in the presence or absence of PGE_2_ (10 nM), PGE_1_-alcohol (1 μM), Pioglitazone (2 μM), T0070907 (100 nM) and MK2206 (0.5 μM) for 30 min. **(H)** ELISA of IgE levels from WT B cells treated with anti-CD40+ IL4 with or without concomitant administration of PGE_1_-alcohol (1 μM) and T0070907 (100 nM) for 7 days (*n* = 3). (mean ± SEM) **(I)**, **(J)** Mice were treated with OVA with or without concomitant administration of PGE_1_-alcohol (500 μg/kg) and T0070907 (500 μg/kg) following a protocol as described in the methods. Serum levels of total **(H)** and OVA-specific **(I)** IgE were determined by ELISA (*n* = 5). (mean ± SD) Data in **(D–G)** are representative data of three independent experiments. Data are pooled from three **(B,C,H–J)** independent experiments. Statistical differences were determined by one-way **(H–J)** ANOVA with Tukey's multiple comparisons test or by unpaired two-tailed Student's *t*-test **(B,C)**. Data are presented as mean ± SD, unless specifically noted. ^*^p < 0.05, ^**^p < 0.01, and ^***^p < 0.001, ns, not significant.

PPARγ is thought to play an important role in the regulation of inflammatory responses. In a murine asthma model, the use of PPARγ agonists can alleviate airway hyperresponsiveness to inhibit the development of allergic inflammation (21). On the other hand, PPARγ ligand 15d-PGJ2 inhibits IL-4 induced IgE class switching in B cells by down-regulating STAT6 phosphorylation. Based on the above, we suspect that PGE_2_-EP4-Akt may inhibit IgE production by PPARγ. We tried to validate our conjecture using the protein-protein interaction network (PPI). The PPI enrichment *p*-value of the pathway EP4, Akt, FoxO1, PPARγ is 0.000834. In addition to PPARγ in this network, other candidates of EP4 were also retrieved, including Hsp90aa1, Nos3, and Mtor (Figure 5A). We tested the levels of these targets using quantitative RT-PCR. No apparent change was found for Hsp90aa1 and Nos3 (Figure 5B); however, mTOR and PPARγ were decreased by varying degrees (Figure 5C). Compared to mild suppressed mTOR mRNA expression, PPARγ mRNA was found to be downregulated by almost 5-fold in EP4^f/f^ Mb1^cre^ B cell after anti-CD40+IL-4 stimulation. Next, we verified the expression of PPARγ at the protein level and a similar decrease in the protein expression of PPARγ was confirmed (Figure 5D). In contrast, the addition of PGE_2_ and EP4 agonist PGE_1_-alcohol can enhance PPARγ expression (Figure 5E). These results show that EP4 signaling can regulate PPARγ expression. In order to explore whether Akt downstream of EP4 signaling can directly regulate PPARγ expression, we used the Akt inhibitor MK2206. As expected, after MK2206 inhibited Akt phosphorylation, PPARγ expression was also down-regulated, suggesting that PPARγ is downstream of Akt signaling (Figure 5F).

Based on this result, it was highly likely that PGE_2_-EP4 signaling regulates IgE production through PPARγ. Therefore, we used a PPARγ agonist (pioglitazone) and antagonist (T0070907) to verify the role of PPARγ in EP4-mediated inhibition *in vitro* and *in vivo*. Our data revealed that PPARγ can manipulate the phosphorylation of STAT6 (Figure 5G) and the consequent IgE production (Figure 5H). Its agonist pioglitazone suppressed phospho-STAT6, while its antagonist T0070907 successfully reversed the inhibition of phospho-STAT6 expression and IgE secretion by PGE_1_-alcohol. Meanwhile, *in vivo* asthma model, the PPARγ antagonist T0070907 was found to boost serum IgE and OVA-specific IgE levels in OVA-induced mice (Figures 5I,J), as well as higher inflammatory infiltration (Figure S3). These effects were very similar to EP4 antagonist ONO-AE3-208 and the findings in EP4-deficient mice. In brief, these data suggest that EP4 further regulates the expression of PPARγ by activating Akt and then PPARγ participates in the down-regulation of phospho-STAT6 and IgE production, eventually attenuating the development of asthma.

### PPARγ Functions as an E3 Ligase to Induce the Ubiquitination of Phospho-STAT6

**Figure 6:**
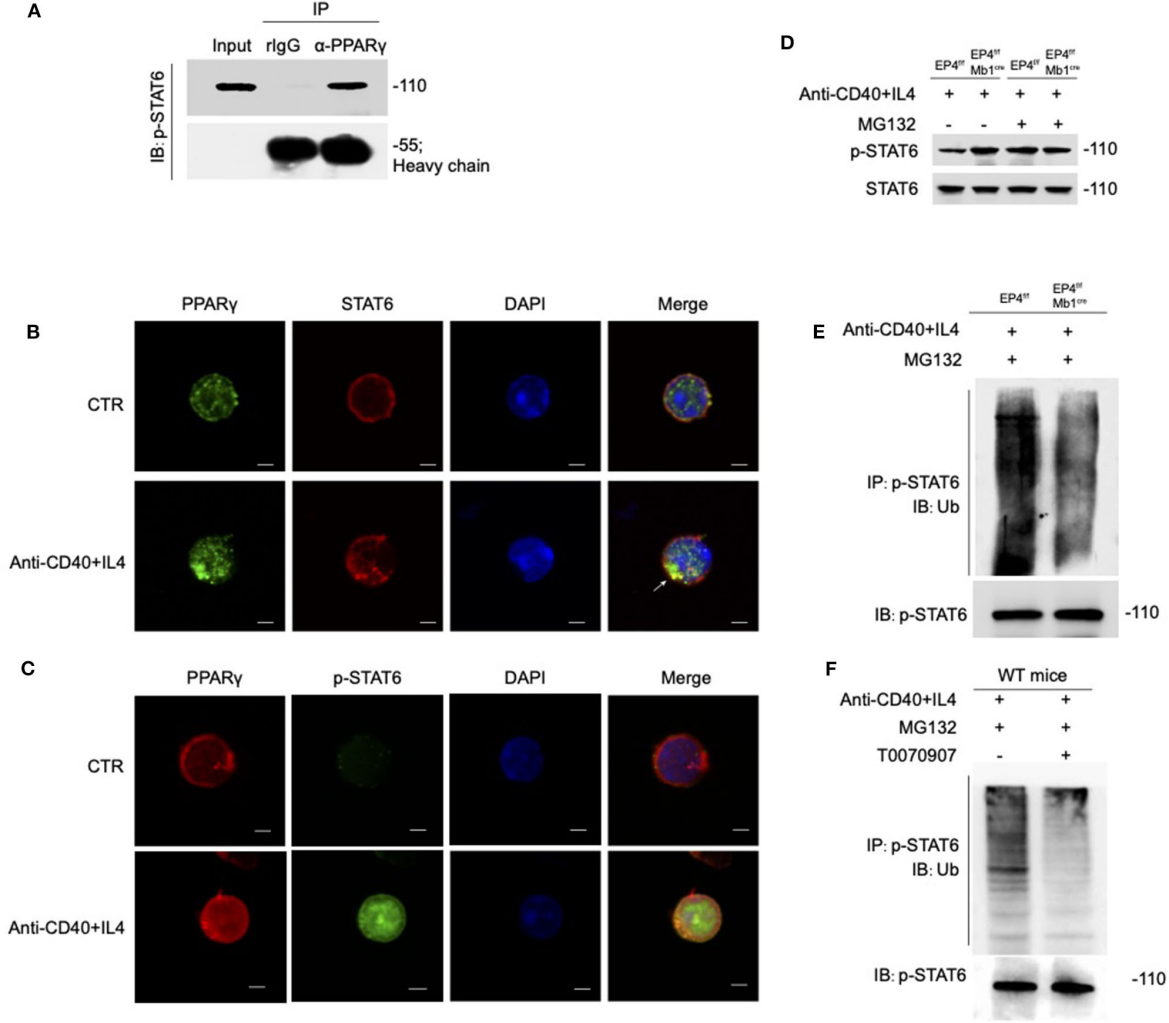
PPARγ induces the ubiquitination of pSTAT6. **(A**) Whole-cell extracts were immunoprecipitated (IP) with anti-PPARγ followed by immunoblotting (IB) with anti-p-STAT6 antibody. Rabbit IgG represents a control antibody used for IP. **(B)** Confocal microscopy of the expression of STAT6 and PPARγ in WT B cells treated with or without anti-CD40+ IL4 for 30 min. Scale bars, 2.5 μm. **(C)** Confocal microscopy of the expression of p-STAT6 and PPARγ immunostaining in the indicated colors in WT B cells treated with or without anti-CD40+ IL4 for 30 min. Scale bars, 2.5 μm. **(D)** EP4^f/f^ and EP4^f/f^ Mb1^cre^ B cells were treated with 20 μM MG132 or DMSO for 10 h before 30 min stimulation of anti-CD40+ IL4 and immunoblotted with indicated antibodies. **(E)** EP4^f/f^ and EP4^f/f^ Mb1^cre^ B cells were treated with 20 μM MG132 for 10 h before 30 min stimulation of anti-CD40+ IL4. IP with anti-p-STAT6 followed by IB with anti-Ubiquitin and anti-p-STAT6 antibody. **(F)** WT B cells were treated with 20 μM MG132 for 10 h before 30 min stimulation of anti-CD40+ IL4 with or without T0070907 (100 nM). IP with anti-p-STAT6 followed by IB with anti-Ubiquitin and anti-p-STAT6 antibody. Each experiment was independently repeated at least three times.

PPARγ is generally thought to play a regulatory role as transcription factors, but recent researches suggest that PPARγ can be an E3 ubiquitin ligase (44–46). According to above results, the overall level of STAT6 has no change, but the phospho-STAT6 has changed. Based on the literature, we speculate that the down-regulation of phosphorylated STAT6 by PPARγ does not occur at the transcription level, but may at the post-translation level. Therefore, we first focused on whether there is a direct interaction between PPARγ and STAT6. As shown in Figure 6A, PPARγ can clearly interact with phospho-STAT6. To further verify their interaction, we used confocal technology to observe the location of the overall and phosphorylated expression of STAT6 and PPARγ in WT B cells. After 30 min of PPARγ treatment with anti-CD40 + IL-4, the green fluorescent spots increased significantly (Figure 6B), indicating that PPARγ can be induced by this stimulation, which is consistent with the previous results (Figure 5E). Also, it can be seen that STAT6 is initially in the cytoplasm, and after activation, it accumulates in the nucleus in large quantities. After merging the layers, we can clearly see the increase of yellow fluorescent spots after stimulation, indicating that PPARγ and total STAT6 are co-localized (Figure 6B). Similarly, we labeled PPARγ and phosphorylated STAT6 in WT B cells and found these two molecules co-localized extensively in the cytoplasm (Figure 6C). This result further supports the results of previous co-immunoprecipitation experiments (Figure 6A).

PPARγ can directly bind to phospho-STAT6, so can PPARγ play its role of E3 ubiquitin ligase to modify ubiquitination of phosphorylated STAT6? We then analyzed the stability of phosphorylated STAT6 upon treatment with the proteasome inhibitor MG132 (Figure 6D). The results showed that without MG132 treatment, the phospho-STAT6 in EP4^f/f^ Mb1^cre^ B cells was significantly increased, but after MG132 treatment, phospho-STAT6 expression in EP4^f/f^ returned to the same level as EP4^f/f^ Mb1^cre^ and the discrepancy between them disappeared, and in either case, total STAT6 was not affected by MG132. It is worth noting that the proteasome inhibitor MG132 can inhibit the degradation of all proteins in the cells and this indiscriminate inhibition leads to the elimination of the original changes in phospho-STAT6. This suggests that changes in phospho-STAT6 result from protein degradation. Furthermore, the ubiquitination level of phospho-STAT6 was immunoprecipitated from B cells pretreated with MG132 and then probed with anti-ubiquitin antibodies. The results showed that the level of ubiquitination modification of phospho-STAT6 in B cell was greatly decreased in EP4^f/f^ Mb1^cre^ cells compared with control cells (Figure 6E). In order to further confirm the role of PPARγ in ubiquitination, this study used the PPARγ antagonist T0070907 on WT B cells. As expected, the inhibition of PPARγ could down-regulate the ubiquitination level of phospho-STAT6 (Figure 6F). Furthermore, compared to control B cells, there was far more phospho-STAT6 and much less PPARγ in EP4^f/f^ Mb1^cre^ B cells after anti-CD40+IL4 stimulation and ubiquitination in locations in which PPARγ and pSTAT6 were expressed together (Figure S4). Thus, these results indicate that PPARγ may physically interact with phospho-STAT6 to modify its proteasome-ubiquitin degradation and help inhibit its activation signal downwards to the nucleus.

Taken together, our data suggested that EP4 receptor promotes the expression of PPARγ by Akt activation, which accelerates the ubiquitination of phosphorylated STAT6. Low-level phosphorylated STAT6 hinders IgE class switching, inhibits IgE production, and ultimately alleviates the development of asthma.

## Discussion

The present study explored the role of EP4-mediated PGE_2_ signaling in the regulation of IgE production and its pathological relevance in asthma. The major findings are briefly summarized as follows: (1) through EP4, PGE_2_ signaling decreases IgE production by inhibiting STAT6 activation and the transcription of downstream targets and attenuates the development of OVA-induced asthma; (2) PPARγ acts as an E3 ubiquitin ligase and induces phospho-STAT6 proteasome-dependent degradation in B cells.

Previously, several analyses of EP-selective agonists have suggested that both the EP2 and EP4 receptors contribute to PGE_2_-induced effects on B cells synergistically (13, 37, 47). However, there was also different voice. *Yuhan Gao* and his colleagues demonstrated that PGE_2_ promotes IgE class switching, and the secretion of IgE by B cells stimulated *in vitro* with LPS+IL4 in an EP2-dependent manner (10). Interestingly, our present study provides evidence that EP4 has a more robust inhibitory effect on IgE production under stimulation with anti-CD40+IL4 than that of EP2. One possible reason for this inconsistency is that different stimuli may have distinct receptor preference. As shown in Figure 2, after treatment with PGE_2_ and anti-CD40+IL4, the expression of EP4 declined probably due to internalization and then activated downstream pathways, not EP2. As we known, LPS, as a bacterial product, leads to T-independent (TI) responses, while anti-CD40 mimics CD40 ligand (CD40L, also known as CD154) expressed by antigen-stimulated T cells and reflects the course of asthma characterized by Th2 cell-mediated responses (4). Therefore, it is possible that TD and TI exposures trigger disparate signaling pathways, but the mechanism behind remains unclear and requires further experiments for elucidation. Another explanation of their controversial role in IgE production may be that these two receptors-triggered pathways are mutually regulated, considering the possible cross-talk between cAMP and Akt. For example, it is reported that Akt-dependent phosphorylation can cause the activation of PDE3B and consequent loss of cAMP-mediated responses (42). We speculate that, if PGE_2_-EP4-Akt pathway is activated together with PKA-cAMP in first when B cells begin to proliferate (48), its suppression on EP2/EP4-cAMP pathway may occur later and, in the end, EP4-Akt pathway will play a dominant role and lead to homeostasis. However, in our hand, we have no evidence of the change of cAMP and CREB, and more experiments are needed to confirm this hypothesis.

In the pathogenesis of asthma, controversy concerning the role of PGE_2_ *in vivo* still exists. One study demonstrated a significant enhancement of IgE in the BALF of OVA-allergic Cox-1- and Cox-2-deficient mice, while it is barely detectable in WT (49). This hypothesis of PGE_2_-mediated protection is also supported by *Zaslona* and his colleagues, who found that PGE_2_ decreases cytokine production and inhibits STAT6 phosphorylation (50). Compared to WT mice, their EP2-deficient mouse model exhibits exaggerated airway inflammation. Moreover, in patients with allergic asthma, inhaled PGE_2_ has been reported to attenuate both early- and late-phase responses to exposure to Ag (51, 52). Another study, however, demonstrated that an EP2-deficient mouse model of OVA-induced asthma exhibits a markedly suppressed IgE antibody response and develop less pronounced airway inflammation (10). This discrepancy may be attributed to the multifaceted activities of PGE_2_ at different stages of asthma development, since the nature of asthma is an extraordinarily heterogeneous disease. Of interest, it has been reported that the predominant EP receptor expressed in the lungs is EP4 (53). However, in the pathogenesis of asthma, the expression of EP4 was decreased compared with that in healthy controls in an analysis of 54 samples (*p* = 0.0003) (accession number GEO: GSE27011). This result is consistent with our observation that EP4 deficiency results in more severe airway inflammation. However, it raises another question: which comes first, asthma or decreased expression of EP4. The answer to this question may determine whether agonists of EP4 are more useful than those of PGE_2_ considering the decrease in EP4 and the diverse effects of PGE_2_. The empirical findings of this study provide a new dynamic view of how PGE_2_ regulates IgE production in asthma development and other than the inhibitory effect on monocytes and eosinophils (54, 55), EP4 signal may have a direct protective impact on IgE secretion.

Another evidence supporting EP4 signal was essential in asthma came from our data that PGE_2_ and PPARγ are closely linked in the context of B cell differentiation and asthma. The association of PGE_2_ with PPARγ is interesting but not surprising. A recent study showed that the EP4 agonist L-902, 688 is able to effectively increase PPARγ expression and attenuate pulmonary arterial hypertension (43). Indeed, we confirmed that EP4 deficiency leads to a decrease in PPARγ expression via immunoblotting and Confocal microscopy. Previously, Miyazaki et al. (56) found that 1 μM 15d-PGJ2, a PPARγ ligand, is able to suppress pSTAT6 and IgE class switching in the human B cell line DND39. However, the mechanism by which PPARγ inhibited pSTAT6 in that study is unclear. Moreover, *Yongzhong Hou* and his colleagues demonstrated that PPARγ is an E3 ligase that induces Lys48-linked ubiquitination and the degradation of nuclear factor-κB (NFkB)/p65 (44). Currently, NFkB/p65, MUC1-C, and SelS, as well as SelK, are known to be ubiquitinated and degraded by PPARγ (45). In our current study, we identified a new target of PPARγ as an E3 ubiquitin ligase in B cells. However, more research on the specific site of PPARγ-mediated ubiquitination in pSTAT6 needs to be conducted in the future.

Notwithstanding these limitations, our study suggests that PGE_2_-inhibited IgE production by B cells is mainly mediated by EP4 through the AKT/FoxO1/PPARγ pathway and PPARγ-mediated ubiquitination on pSTAT6. Given PPARγ agonists have been reported to treat inflammatory lung disease (43, 57), EP4 regulation combined with PPARγ may present a potential therapeutic strategy for asthma in future.

## Data Availability Statement

Publicly available datasets were analyzed in this study. This data can be found here: GEO: GSE27011.

## Ethics Statement

The animal study was carried out in accordance with the recommendations of the Ethics Committee of Peking University Health Science Center. The protocol (No. LA2018106) was approved by the Ethics Committee of Peking University Health Science Center.

## Author Contributions

JW, WW, and YZhang designed the project. JW and WW did the experiment and wrote the manuscript. YG hybrid the mice. YaW, YZhou, and YuW contributed to establish the asthma model and analyze the data. XS and YZhao contributed to Confocal fluorescence microscopy examination. All authors contributed to the article and approved the submitted version.

## Conflict of Interest

The authors declare that the research was conducted in the absence of any commercial or financial relationships that could be construed as a potential conflict of interest.

## Supplementary Material

Figure S1 The EP4 deficiency makes no difference to normal development of B cells. **(A)** B220^+^B cell ratio of bone marrow (*n* = 20). **(B)** The cell number of subgroups in bone marrow using the following markers: proB (B220^+^CD43^+^), preB (B220^+^CD43^−^IgM^low^), immature B (B220^+^CD43^−^IgM^hi^), mature B (B220^hi^CD43^−^IgM^hi^) (*n* = 5). **(C)** B220^+^B cell count of spleen, lymph nodes (*n* = 20). **(D)** The cell number of subgroups of T1B cells (B220^+^CD23^−^CD21/35^−^IgM^−^), T2B cells (B220^+^CD23^+^CD21/35^hi^IgM^hi^), marginal zone (MZ) B cells (B220^+^CD23^−^CD21/35^+^IgM^+^), follicular (FO) B cells (B220^+^CD23^+^CD21/35^low^IgM^low^) (*n* = 5). **(E)** The expression of activation marker of primary B cells from EP4^f/f^ and EP4 KO mice were examined by flow cytometry. Data are presented as mean ± SD; ns, not significant.

Figure S2 The effects of EP4 agonist and antagonist on the OVA-induced asthma model. **(A)** WT were immunized following a protocol as described in the methods. **(B)** Total cell number in the BALF collected 24h after the last challenge (n = 7). **(C)** Representative images showing HE and PAS staining of the lung tissue. Scale bar equals 100 μm (upper) or 50 μm (down). Data are presented as mean ± SD. Data are pooled from three independent experiments. ^*^*p* < 0.05; ^**^*p* < 0.01; ns, not significant.

Figure S3 The effects of EP4 agonist and PPARγ antagonist on the OVA-induced asthma model. **(A)** WT were immunized following a protocol as described in the methods. **(B)** Total cell number in the BALF collected 24h after the last challenge (*n* = 5). **(C)** Representative images showing HE and PAS staining of the lung tissue. Scale bar equals 100 μm (upper) or 50 μm (down). Data are presented as mean ± SD. Data are pooled from three independent experiments. ^**^*p* < 0.01; ^***^*p* < 0.001; ns, not significant.

Figure S4 Confocal microscopy of the expression of p-STAT6, PPARγ and ubiquitin in WT B cells. Confocal microscopy in EP4^f/f^ and EP4 KO B cells treated with anti-CD40^+^ IL4 for 30 min. Scale bars, 2.5 μm. Data are representative data of three independent experiments.
