# Supplementary figures and images for: PPARγ as an E3 Ubiquitin-Ligase Impedes Phosphate-Stat6 Stability and Promotes Prostaglandins E2-Mediated Inhibition of IgE Production in Asthma
Source: Front Immunol. 2020 Jun 19;11:1224. doi: 10.3389/fimmu.2020.01224 (PMC7317005; doi:10.3389/fimmu.2020.01224)

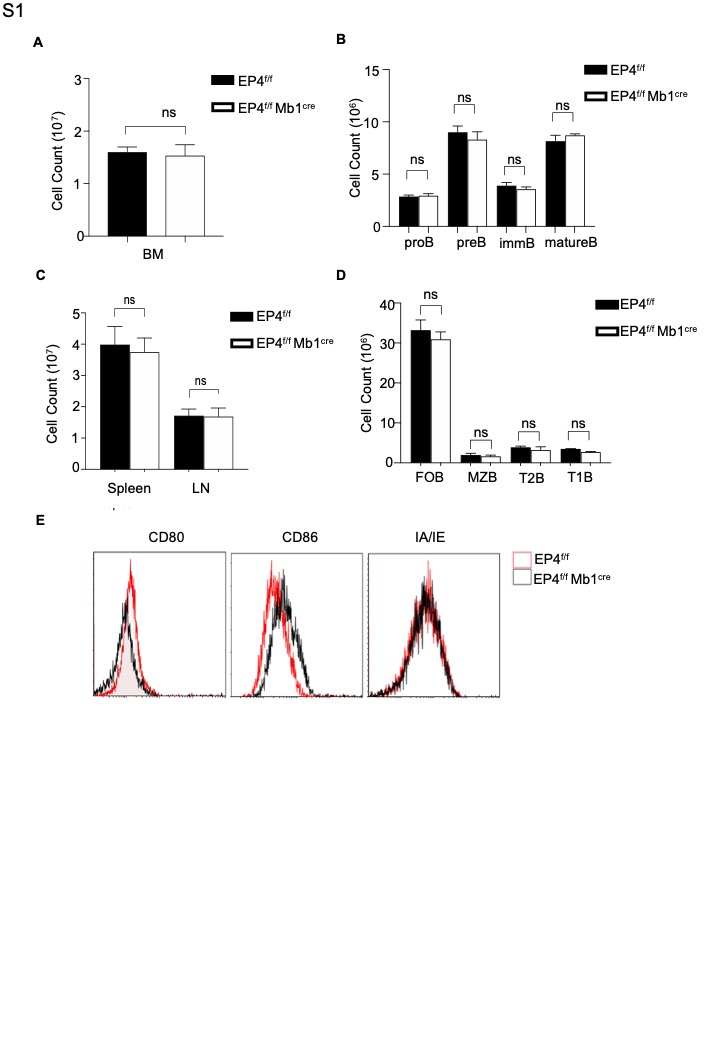

Supplement: Figure S1 — The EP4 deficiency makes no difference to normal development of B cells. (A) B220+B cell ratio of bone marrow (n = 20). (B) The cell number of subgroups in bone marrow using the following markers: proB (B220+CD43+), preB (B220+CD43−IgMlow), immature B (B220+CD43−IgMhi), mature B (B220hiCD43−IgMhi) (n = 5). (C) B220+B cell count of spleen, lymph nodes (n = 20). (D) The cell number of subgroups of T1B cells (B220+CD23−CD21/35−IgM−), T2B cells (B220+CD23+CD21/35hiIgMhi), marginal zone (MZ) B cells (B220+CD23−CD21/35+IgM+), follicular (FO) B cells (B220+CD23+CD21/35lowIgMlow) (n = 5). (E) The expression of activation marker of primary B cells from EP4f/f and EP4 KO mice were examined by flow cytometry. Data are presented as mean ± SD; ns, not significant. [file Image_1.jpeg]

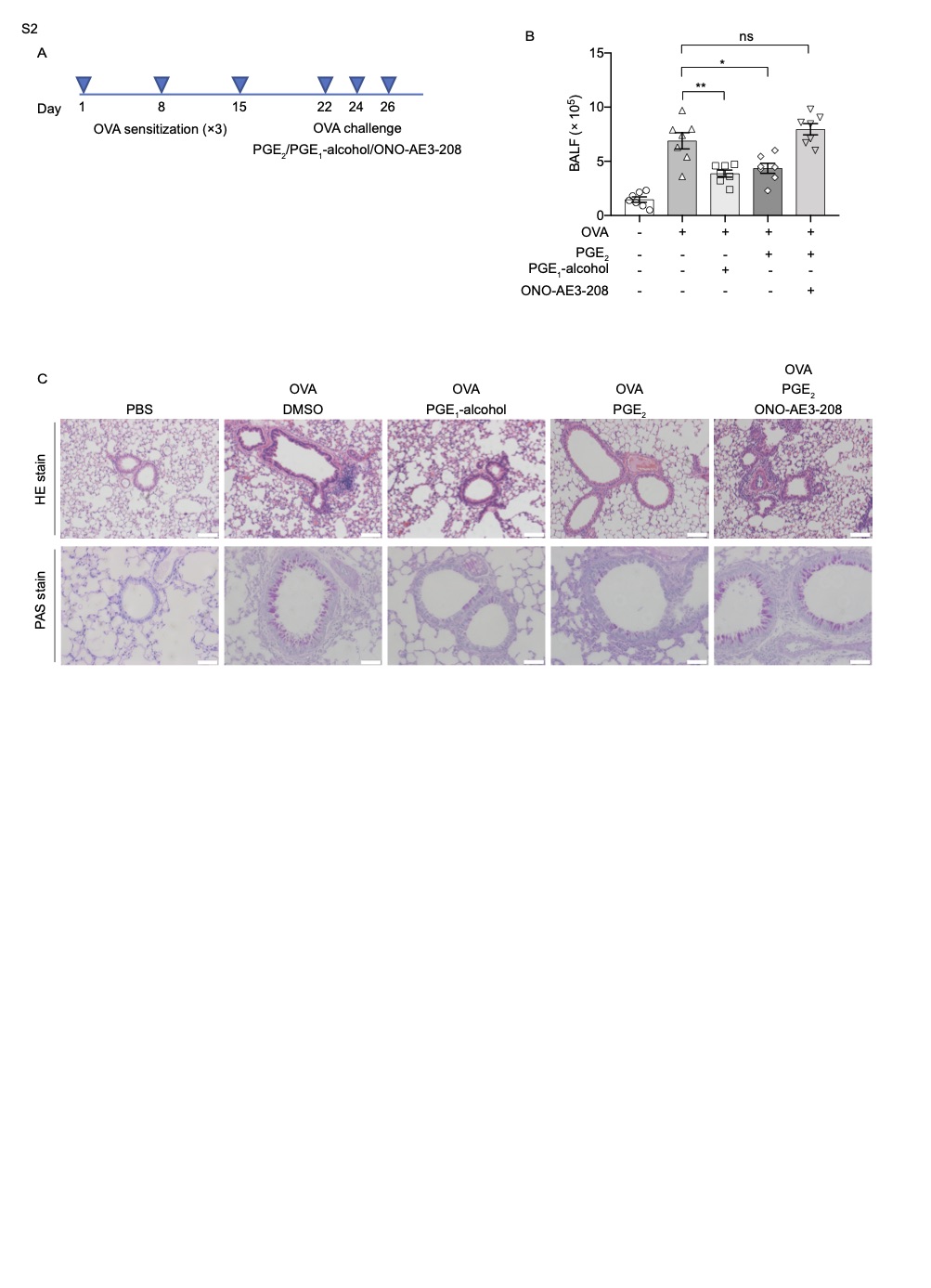

Supplement: Figure S2 — The effects of EP4 agonist and antagonist on the OVA-induced asthma model. (A) WT were immunized following a protocol as described in the methods. (B) Total cell number in the BALF collected 24h after the last challenge (n = 7). (C) Representative images showing HE and PAS staining of the lung tissue. Scale bar equals 100 μm (upper) or 50 μm (down). Data are presented as mean ± SD. Data are pooled from three independent experiments. *p < 0.05; **p < 0.01; ns, not significant. [file Image_2.jpeg]

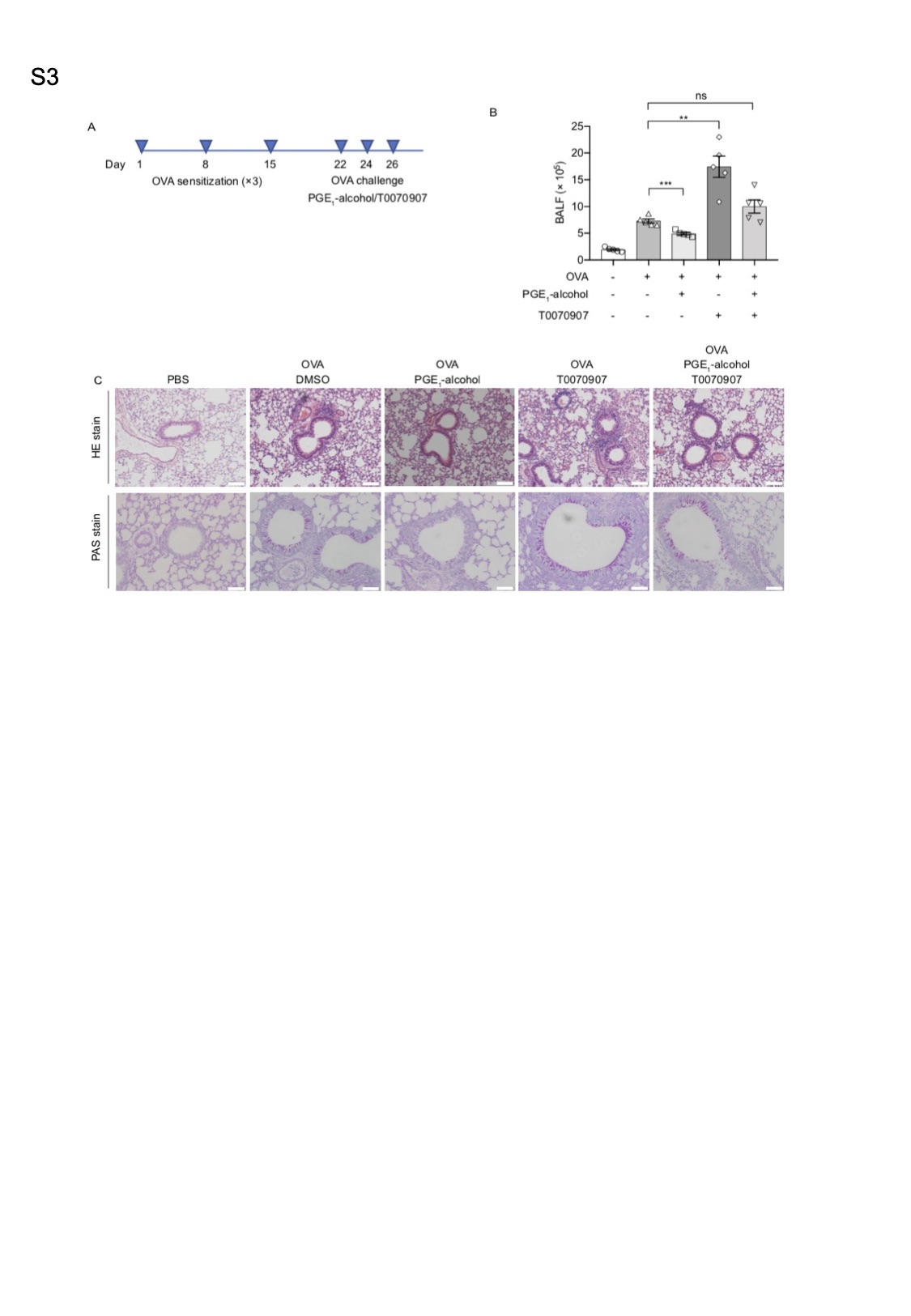

Supplement: Figure S3 — The effects of EP4 agonist and PPARγ antagonist on the OVA-induced asthma model. (A) WT were immunized following a protocol as described in the methods. (B) Total cell number in the BALF collected 24h after the last challenge (n = 5). (C) Representative images showing HE and PAS staining of the lung tissue. Scale bar equals 100 μm (upper) or 50 μm (down). Data are presented as mean ± SD. Data are pooled from three independent experiments. **p < 0.01; ***p < 0.001; ns, not significant. [file Image_3.jpeg]

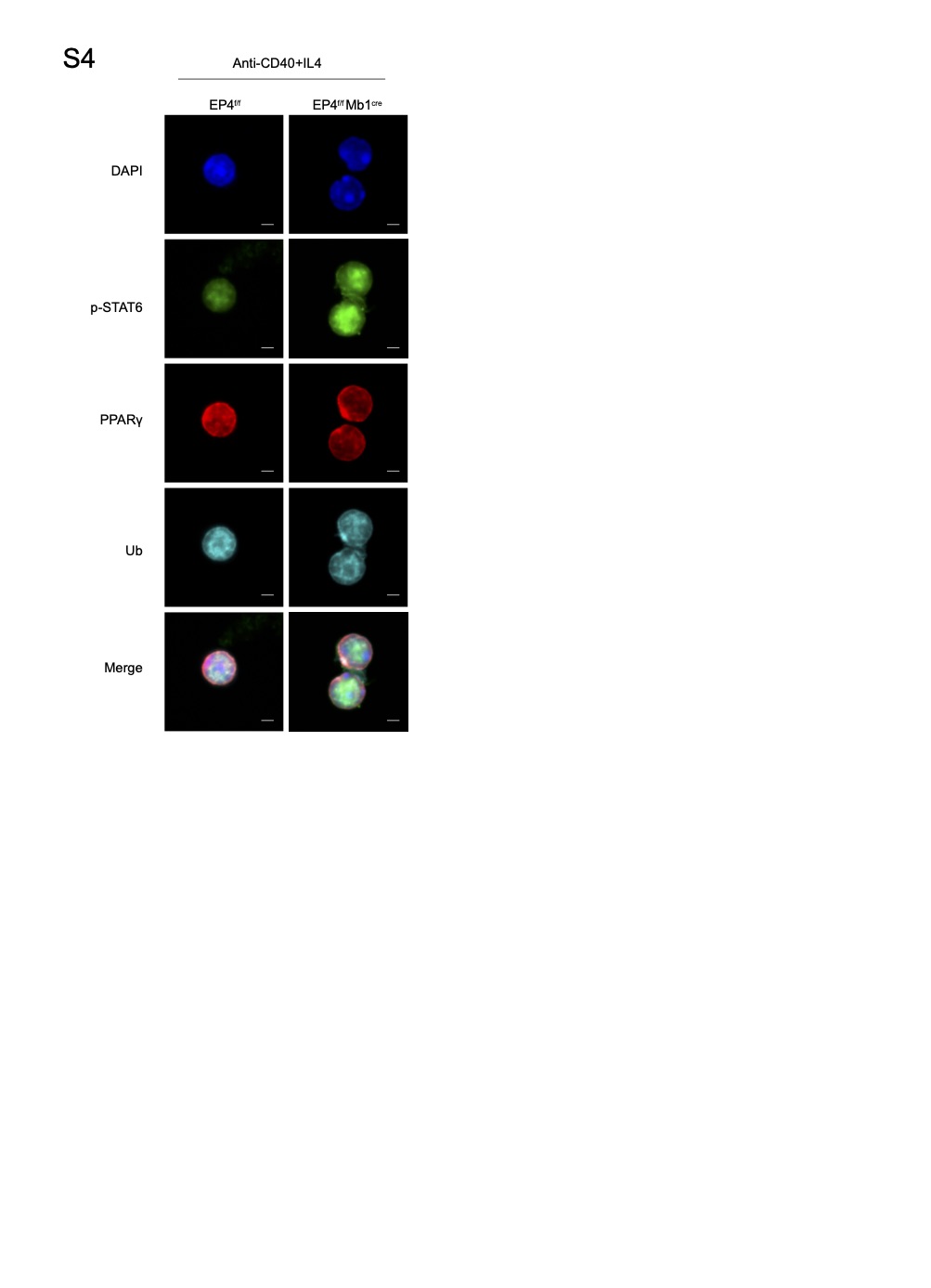

Supplement: Figure S4 — Confocal microscopy of the expression of p-STAT6, PPARγ and ubiquitin in WT B cells. Confocal microscopy in EP4f/f and EP4 KO B cells treated with anti-CD40+ IL4 for 30 min. Scale bars, 2.5 μm. Data are representative data of three independent experiments. [file Image_4.jpeg]
